# Synergistic antibacterial activity of silver nanoparticles biosynthesized by carbapenem-resistant Gram-negative bacilli

**DOI:** 10.1038/s41598-022-19698-0

**Published:** 2022-09-09

**Authors:** Sayran Hamad Haji, Fattma A. Ali, Safaa Toma Hanna Aka

**Affiliations:** 1 Department of Pharmacognosy, College of Pharmacy, Hawler Medical University, Erbil, Iraq; 2 Department of Medical Microbiology, College of Health Science, Hawler Medical University, Erbil, Iraq

**Keywords:** Microbiology, Molecular biology

## Abstract

Nanotechnology is being investigated for its potential to improve nanomedicine for human health. The purpose of this study was to isolate carbapenemase-producing Gram-negative bacilli (CPGB), investigate the presence of carbapenemase resistance genes, determine their antibiogram and ability to biosynthesise silver nanoparticles (Ag NPs), and estimate the antibacterial activity of *Acinetobacter baumannii*-biosynthesised Ag NPs on CPGB alone and in combination with antibiotics. A total of 51 CPGBs were isolated from various specimens in the study. The automated Vitek-2 system was used to identify and test these strains' antimicrobial susceptibilities. The carbapenemase resistance genes were identified using a polymerase chain reaction (PCR). Under the CPGB, *A. baumannii* could biosynthesise Ag NPs. X-ray diffraction (XRD), Fourier-transform infrared spectroscopy (FT-IR), transmission electron microscopy (TEM), and field emission scanning electron were used to characterise Ag NPs. The antibacterial activity of Ag NP alone and in combination with antibiotics against CPGB was determined using the broth microdilution method, and their synergistic effect was determined using the checkerboard assay. *bla*_NDM_ and *bla*_OXA-48_ were the most commonly reported, and 90% of the isolates produced multiple carbapenemase genes. Tigecycline proved to be the most effective anti-CPGB antibiotic. Isolates with more resistance genes were more resistant to antibiotics, and isolates with three genes (42%) had the most extensively drug-resistant patterns (38%). A significant relationship was discovered between genetic and antibiotic resistance patterns. Only *A. baumannii* produced Ag NPs out of all the isolates tested. Ag NPs with a size of 10 nm were confirmed by UV–visible spectroscopy, FT-IR, XRD, and TEM analysis. The Ag NPs were effective against CPGB, with minimum inhibitory concentrations ranging from 64 to 8 μg/ml on average. Surprisingly, the combination of Ag NPs and antibiotics demonstrated synergistic and partial synergistic activity (fractional inhibitory concentration between 0.13 and 0.56) against CPGB, as well as a significant reduction in antibiotic concentrations, particularly in the case of *A. baumanii* versus ceftriaxone (1024 to 4 μg/ml). The notable synergistic activity of Ag NPs with antibiotics represents a valuable nanomedicine that may find clinical application in the future as a combined remedy.

## Introduction

Nanotechnology is a modern branch of biotechnology concerned with the synthesis, design, and processing of nanoscale particles ranging in size from 1 to 100 nm^1^. The biosynthesis of nanoparticles (NPs) by bacteria, plants, fungi, or algae serves as a potential 'nanofactory' for the development of safe, non-toxic, cost-effective, and environmentally friendly technologies for a variety of applications in basic and applied sciences^2^. NPs have a large surface area to volume ratio and a high number of surface atoms, which have been extensively studied for their unique chemical and physical properties, including magnetic, electronic, optical, antimicrobial properties, and catalytic activity^3^.

NPs are created using various chemical and physical methods^4^. Green microbial synthesis has recently been recognised as a promising resource capable of significantly overcoming the ecological challenges of chemical and physical methods that do not involve hazardous chemicals and intolerant interaction conditions^5^. Among the various biological entities, bacterial NP synthesis is preferred due to its large production. Bacterial NP synthesis is preferred due to its high output, rapid growth rate, and ease of control^6^. Multidrug-resistant, community-acquired, and hospital-acquired bacterial infections are on the rise worldwide, with few active treatment options available. One prominent example is the rise in the prevalence of carbapenem-resistant *Enterobacteriaceae*. To make matters worse, there is no viable alternative treatment for this extremely resistant infection. As a result, antimicrobial resistance has increased mortality and morbidity rates for potentially life-threatening bacterial infections. Similarly, antimicrobial resistance has become one of the greatest threats to human health^7^.

Since 1993, various types of carbapenemases belonging to 3 molecular classes have been recognised, namely Ambler classes A, B and D beta-lactamases. Class A carbapenemases, Klebsiella pneumoniae carbapenemase (KPC); Class B Metallo-beta-lactamases (MBLs) e.g., Verona Integron-encoded Metallo-beta-lactamase (VIM), New Delhi Metallo-beta-lactamases (NDM), and Imipenemase (IMP); Class D carbapenem-hydrolysing oxacillinase (OXA) for instance OXA-48^8^.

According to recent research, antibiotic resistance in bacteria is spreading faster than antimicrobial agents to combat it. As a result, there is a constant increase in interest in the synthesis of new products with antimicrobial potential. The same is true for silver, a metal that, in addition to silver nanoparticles (Ag NPs), is thought to have antibacterial activity and significant efficacy against gram-positive and gram-negative bacteria^9^. This area of research should become a challenge for biotechnology and nanomedicine. This field of study should pose a challenge to biotechnology and nanomedicine. Antibiotics are also thought to be transported by Ag NPs, facilitating bacterial cell permeation and increasing antibacterial activity. Furthermore, combining antibiotics with Ag NP reduces both compounds' toxicity to human cells by lowering the desired dose while increasing antimicrobial properties^10^.

However, in spite of numerous studies, the present available information is not sufficient to confirm the adverse effects of nanoparticle-antimicrobials on human health. Therefore, more research needs to be done to mitigate any toxicity issues that may arise^11^.

The emergence of pan drug-resistant (PDR), extensively drug-resistant (XDR), and multidrug-resistant (MDR) bacteria, particularly Gram-negative bacteria resistant to multiple drugs, delays the efficacy of many antimicrobial agents. Isolates were classified as MDR if they were resistant to at least three commonly used and effective antimicrobial classes. XDR bacteria were resistant to all but two or fewer classes of antimicrobials, whereas PDR bacteria were resistant to all available antimicrobial classes^12,13^.

There will be a serious risk and widespread clinical problem in the near future because there will be few or no antimicrobials available for drug-resistant pathogens^14^. As a result, alternative strategies to antibacterial drugs or pharmaceutical combinations that block resistance mechanisms while treating microbial diseases are critical. The use of various metallic NPs is one such strategy^15^.

The purpose of this study was to isolate carbapenemase-producing gram-negative bacilli (CPGB), screen for carbapenemase resistance genes, determine their antibiogram and ability to biosynthesise Ag NPs, characterise the synthesised NPs, and evaluate their antibacterial activity against CPGB alone and in combination with beta-lactam antibiotics.

## Materials and methods

### Bacterial isolates

This study included 51 genotype-confirmed clinical isolates of CPGB that demonstrated MDR to several antibiotic classes tested, including carbapenems. These isolates were identified using traditional bacteriological and biochemical techniques as well as the automated Vitek-2 system (bioMerieux, USA) (GN-card)^16^. Between February and September 2020, all isolates were collected from patients in hospitals and communities in Erbil, Iraq's Kurdistan Region. The isolated strains were obtained from various infection sites, including urine (24), sputum (16), wound swabs (8), and blood (3). The ethics committee of Hawler Medical University's College of Pharmacy approved this study. Written informed consent was obtained from the participants. All methods were performed in accordance with relevant guidelines and regulations. During data collection, all patients who attended treatment were asked to obtain verbal consent. The purpose of the study was explained to the participants and details were provided about anonymity and the right to withdraw from the study at any stage. Finally, verbal consent was obtained from the participants in this research.

### Antibacterial susceptibility test

The antimicrobials tested were piperacillin and piperacillin-tazobactam (β-lactams), ceftriaxone, ceftazidime, cefazolin and cefepime (cephalosporins), gentamicin and amikacin (aminoglycosides), ciprofloxacin (quinolone), imipenem, ertapenem and meropenem (carbapenems), trimethoprim / sulfamethoxazole (sulfonamides), nitrofurantoin, and tigecycline (tetracycline). The susceptibility of CPGB was tested using the Vitek-2 automated system according to the manufacturer's instructions^17^. Phenotypic resistance profiles are classified as XDR, PDR, and MDR as defined by Algammal^12^.

### Molecular analysis of carbapenemase genes

Using a set of primers previously described^18–20^, a polymerase chain reaction (PCR) was used to screen carbapenemase genes, MBLs (*bla*_NDM_, *bla*_VIM_, and *bla*_IMP_ genes), OXA-48 (*bla*_OXA-48_ gene), and KPC (*bla*_KPC_ gene) under CPGB. Multiplex PCR was used to detect the presence of *bla*_VIM_ and *bla*_IMP_. Uniplex PCR was used to examine the *bla*_OXA-48_, *bla*_KPC_, and *bla*_NDM_ genes. Total DNA was extracted from bacterial cultures in the logarithmic phase using a commercial genomic DNA extraction kit (DNAL and Scientific Cat No. GG2001) and the manufacturer's instructions. The following PCR amplification steps were carried out on a thermocycler machine (Techne, UK): The premix was exposed to a total volume of 25 μl, 12.5 μl Gotaq Green Master Mix (Promega/USA), 3 μl genomic DNA, 1.5 μl of each primer, and 6.5 μl of nuclease-free water for uniplex PCR. In multiplex PCR, a total volume of 25 μl was used, with 12.5 μl of Gotaq Green Master Mix (Promega/USA), 3 μl of genomic DNA, 1 μl of each primer, and the volume supplemented with 5.5 μl of DNase and RNase-free water. The amplification conditions for these genes were set as previously described^3^.

### Ag NP biosynthesis

The biosynthesis of Ag NPs was carried out using the previously described method^5^. To investigate the biosynthesis of Ag NPs, all CPGBs were used. Only *A. baumannii* isolates produced positive results for Ag NP synthesis. The *Acinetobacter baumannii* isolate was first cultured in a sterile flask containing 100 ml of trypticase soy broth (TSB) medium (Oxoid). The culture flask was incubated at 35 °C on an orbital shaker (120 rpm). After 24 h, the culture was centrifuged at 1,000 rpm for 10 min, and the supernatant was mixed with a 10 mM silver nitrate (AgNO3) solution (Sigma-Aldrich, USA, 99.9%) (1:1, v/v) and incubated at 35 °C in the dark for one day. The TSB medium mixed with culture supernatant was used as a control (without adding AgNO3)^1^. After obtaining the Ag NPs, they were dried overnight at 60 °C for further characterisation and applications^21^.

### Characterization of Ag NPs

The NP formation was tracked by changing the colour of the culture supernatant from light yellow to dark brown. This was confirmed by using a dual-beam UV–visible spectrophotometer to measure the peak exhibited by the Ag NPs (Perkin Elmer Lambda, 35USA). The UV–visible spectra of aliquots of culture supernatant (2 ml) were recorded in the wavelength range of 300–800 nm, with double distilled water serving as a blank^5,22^. Fourier-transform infrared spectroscopy (FT-IR) was used to investigate the properties of the functional groups in Ag NPs (Spectrum 4600 from JASCO). The NP solution was analysed in the 4000–400 cm^−1^ spectral range with a resolution of 4 cm^−1^^5^. The crystalline structure and chemical composition of Ag NP were determined using XRD measurements (PAN analytical X'Pert PRO) equipped with a Cu K (k = 1.5406) radiation. The instrument was set to 40 mA and 40 kV, and the diffraction intensities were recorded in the 2θ range of 20°–80°. On the XRD grid, dry powder samples of Ag NP were deposited^21^. Advanced transmission electron microscopy (TEM; Titan3 G2 60–300, Cs corrector: image and probe, FEI, USA) and field emission scanning electron (FESEM) were used to study the particle size distribution and morphology of Ag NP (Quanta 4500). The sample was prepared as a thin film by drop casting on a grid coated with Cu TEM holey carbon and air dried^23^. Energy dispersive X-ray spectroscopy (EDX) in conjunction with a FESEM was used to investigate the chemical composition of the Ag NPs. On a carbon-coated copper grid, the samples were prepared and dried^1,22^.

### Antibacterial susceptibility of Ag NPs

#### Determination of minimum inhibitory concentration and minimum bactericidal concentration of Ag NPs and antibiotics of clinical pathogens

The MIC of the biosynthesized silver NPs against clinical isolates of CPGB were examined using the broth microdilution method according to a conventional method^10,24^. Biosynthesized silver NPs were serially diluted in sterile Muller Hinton Broth (MHB) (Oxoid) at an initial concentration of 1,024 to 1 μg/ml. Each well was then inoculated with 100 µl of 0.5 McFarland (10^8^ CFU/ml of medium) of the bacterial suspension for each isolate to obtain a final volume of 200 µl in 10 replicates. Final silver concentrations 1, 2, 4, 8, 16, 32, 64, 128, 256, 512, and 1024 μg/ml. MHB wells with inoculated and sterile uninoculated bacteria were used as a positive and negative control, respectively. Microtiter plates were incubated at 37 °C for 24 h. In order to investigate the lowest concentration of NPs that inhibit bacterial growth, the minimal inhibitory concentration was recorded at which no visible growth was observed.

MIC of the clinical pathogens to antibacterial agents was determined by using broth microdilution method^25^. In this study, imipenem from ChemCruz-USA, ceftazidime, ceftriaxone, and cefepime from Awamedica-Iraq were used as antimicrobials.

Standard suspension of bacterial pathogens (5 × 10^8^ cell/ml) was added to microdilution wells containing 100 μL MHB and different concentrations of imipenem, ceftazidime, ceftriaxone, and cefepime, (0.125, 0.25, 0.5, 1, 2, 4, 8, 16, 32 and 64 μg/ml) for imipenem, and (1, 2, 4, 8, 16, 32, 64, 128, 256, 512, and 1,024 μg/ml) for ceftazidime, ceftriaxone, and cefepime were prepared. Two wells containing antibiotic and broth served as negative control and positive control, respectively. After 24 h incubation at 37C°, the tubes were examined for growth. The lowest concentration that showed no growth was expressed as the MIC of antibiotics. The concentration displayed no bacterial colony on Mueller–Hinton agar plates after the incubation period was considered to be the lowest bactericidal concentration. Experiments were carried out in triplicates.

### Tolerance level

The tolerance levels of selected CPGB against Ag NPs and antimicrobials were determined according to the technique of Das et al.^26^, using the following formula: Tolerance = MBC / MIC.

### Evaluation of the synergistic test between Ag NP and antibiotics against clinical pathogens

As previously described, a checkerboard titration method was used on standard 96-well microtitre plates to determine the fractional inhibitory concentration indexes (FICI) of each antibacterial compound. The degree of antimicrobial synergy is frequently expressed in terms of FIC^27,28^. FIC is calculated by dividing the MIC for a drug in combination by the MIC for drugs acting alone. Four beta-lactam antibiotics, including carbapenems and cephalosporins, namely imipenem, ceftazidime, ceftriaxone, and cefepime, were tested at MIC levels with prepared Ag NPs against the selected MDR–CPGB. Stock solutions of Ag NPs and antibiotics with sub-MIC values were prepared using this method. Each microtitre well received a total of 100 μL of MHB medium. 50 μL of Ag NP and antibiotic solutions were inoculated into each well of one microtitre plate and serially diluted along the ordinate, while the second was diluted along the abscissa. Add 100 μL CPGB into each well, equivalent to a 0.5 MacFarland suspension (total volume per well: 200 μL). The MICs of the antibiotics and Ag NP in combination were determined after 24 h of incubation at 37 °C, and the FIC was calculated as follows: Antibiotic A FIC = MIC of antibiotic A in combination/MIC of antibiotic A alone; antibiotic B FIC = MIC of antibiotic B in combination/the MIC of antibiotic B alone; the FICI = FIC of antibiotic A plus the FIC of antibiotic B. The FICI calculated was defined as follows: FICI ≤ 0.5 synergistic effect, 0.5 < FICI < 1 partial synergistic effect, FICI = 1 additive, 2 ≤ FICI < 4 indifferent, and 4 < FICI antagonism^25,27,28^.

### Statistical analysis

GraphPad Prism (version 5; GraphPad Software, San Diego, CA). The chi-square test was used to analyse the data collected. A statistically significant P-value of 0.05 was used.

## Results and discussion

### Species identification

From various clinical specimens, a total of 51 identified CPGBs were obtained. Non-fermenting microorganisms and members of the *Enterobacteriaceae* family were found in the isolates, including *Klebsiella* sp. (*K. pneumoniae*) (n = 12, 23%), *Acinetobacter baumannii* (n = 11, 21%), *Escherichia coli* (n = 10, 19%), *Pseudomonas aeruginosa* (n = 8, 15%), and *Proteus* sp. (*P*. *mirabilis*) (n = 3, 5%). Fewer predominant microorganisms, such as *Serratia marcescens*, *Enterobacter cloacae*, *Stenotrophomonas maltophilia*, *Morganella morganii*, *Achromobacter sp.*, *Sphingomonas sp.* and *Pantoea sp.*, had one isolate each (n = 1, 1.9%). Isolates were primarily isolated from urine (47%), sputum (31%), swabs (15%), and blood (5%) (Table 1). Statistical analysis revealed that the distribution of CPGB samples differed significantly from CPGB (P < 0.0001). Microbiological analysis revealed that *K. pneumoniae* (23%) was the most common CPGB recovered from the various clinical specimens, followed by *A. baumannii* (21%). These findings are consistent with those of Bourafa et al. and Codjoe^29,30^.

**Table 1:** Samples distribution and sensitivity rate of 51 CPGB.

Types of bacteria and number of tested isolates (%)	Specimens	Antibiotics and the number of resistance strains (%)														
		*PIP	PTZ	CZ	CRO	CFP	CAZ	IMP	MEM	ERT	AK	GM	CIP	NFN	SXT	TGC
*Klebsiella* n = 12 (23)	7 Urines, 4 Sputum, 1 Swab	12	6	12	12	12	12	4	1	_	3	8	10	6	8	2
		(100)	(50)	(100)	(100)	(100)	(100)	(33)	(8)		(25)	(66)	(83)	(50)	(66)	(16)
*Acinetobacter sp.* n = 11 (21)	1 Urine, 9 Sputum, 1 Swab	11	11	11	11	11	11	11	11	11	11	11	11	11	11	9
		(100)	(100)	(100)	(100)	(100)	(100)	(100)	(100)	(100)	(100)	(100)	(100)	(100)	(100)	(81)
*E. coli* n = 10 (19)	8 Urines, 1 Blood, 1 Swab	10	7	10	10	10	10	1	1	1	5	5	6	2	8	2
		(100)	(70)	(100)	(100)	(100)	(100)	(10)	(10)	(10)	(50)	(50)	(60)	(20)	(80)	(21)
*Pseudomonas sp.* n = 8 (15)	5 Urines, 1Sputum, 2 Swabs	8	4	8	7	8	4	4	4	4	5	6	5	7	4	2
		(100)	(50)	(100)	(87)	(100)	(50)	(50)	(50)	(50)	(62)	(75)	(62)	(87)	(50)	(25)
*Proteus* n = 3 (5)	1 Urine, 1 Sputum, 1 Swab	3	1	3	3	2	2	3	_	1	1	1	2	2	2	1
		(100)	(33)	(100)	(100)	(66)	(66)	(100)		(33)	(33)	(33)	(66)	(66)	(66)	(33)
*Serratia* n = 1 (1.9)	1 Sputum	1	0	1	1	1	1	1	1	1	1	1	0	1	1	0
		(100)		(100)	(100)	(100)	(100)	(100)	(100)	(100)	(100)	(100)		(100)	(100)	
*Enterobacter sp.* n = 1 (1.9)	1 Swab	1	1	1	1	1	1	1	1	1	1	1	1	1	1	1
		(100)	(100)	(100)	(100)	(100)	(100)	(100)	(100)	(100)	(100)	(100)	(100)	(100)	(100)	(100)
*Stenotrophomonasaas sp.* n = 1 (1.9)	1 Blood	1	1	1	1	1	1	1	1	1	1	1	1	1	1	1
		(100)	(100)	(100)	(100)	(100)	(100)	(100)	(100)	(100)	(100)	(100)	(100)	(100)	(100)	(100)
*Morganella sp.* n = 1 (1.9)	1 Urine	0	0	1	0	0	0	0	0	0	0	1	1	1	1	0
				(100)								(100)	(100)	(100)	(100)	
*Achromobacter sp.* n = 1 (1.9)	1 Swab	0	0	0	0	0	0	0	0	0	0	1	0	0	0	0
												(100)				
*Sphingomonas sp.* n = 1 (1.9)	1 Blood	1	1	1	1	1	1	1	1	1	1	1	1	1	1	1
		(100)	(100)	(100)	(100)	(100)	(100)	(100)	(100)	(100)	(100)	(100)	(100)	(100)	(100)	(100)
*Pantoea* n = 1 (1.9)	1 Urine	1	0	0	1	-	-	0	1	0	0	1	0	0	0	0
		(100)			(100)				(100)			(100)				
Total number of isolates (%)	24 Urines (47) 16 Sputum (31)	49	32	49	48	47	43	27	22	21	29	38	38	33	38	19
	8 Swabs (15) 3 Blood (5)	(96)	(62)	(96)	(94)	(92)	(84)	(52)	(43)	(41)	(56)	(74)	(74)	(64)	(74)	(37)
P-value < 0.0001																

*Piperacillin (PIP), piperacillin-tazobactam (TPZ), ceftriaxone (CRO), ceftazidime (CAZ), cefazoline (CZ), cefepime (FEP), gentamicin (GM), amikacin (AK), ciprofloxacin (CIP), imipenem (IMP), ertapenem (ERT), meropenem (MEM), trimethoprim/sulphamethoxazole (SXT), nitrofurantoin (NFN), tigecycline (TGC).

### Results of the antimicrobial sensitivity profile

Table 1 shows the antibiogram investigation of CPGB against the 15 antimicrobials tested. The isolates' resistance patterns revealed high rates of resistance to the antimicrobials tested. The findings revealed that 92–96% of the strains tested positive for resistance to cefazolin, ceftriaxone, cefepime, and piperacillin. Ceftazidime, piperacillin-tazobactam, amikacin, imipenem, meropenem, ertapenem, and nitrofurantoin resistance rates were 84%, 62%, 56%, 52%, 43%, 41%, and 64%, respectively. Ciprofloxacin, gentamicin, and trimethoprim/sulfamethoxazole resistance were found in 74% of the isolates. Tigecycline had the lowest rate of resistance, at 37%. The sensitivity rate of isolates demonstrated a significantly higher difference in resistance to several antimicrobials tested (p < 0.0001) among CPGB (Table 1). Resistance to penicillins, cephalosporins, quinolones, sulfonamides, and aminoglycosides was increased in CPGB. Resistance rates for imipenem, meropenem, and ertapenem were 52%, 43%, and 41%, respectively. The emergence of these resistant strains has resulted in concerning health issues. When CPGB was compared to other MDR isolates, it showed remarkable antimicrobial resistance. According to other studies, the pattern of imipenem resistance in *A. baumannii* in this study was (100%)^29,31^, and higher than what Zendegani and Dolatabadi^32^ discovered. They discovered that 76% of the 100 *A. baumannii* isolates tested positive for imipenem resistance. Furthermore, CPGB was susceptible to tigecycline, affecting approximately 65% of MDR isolates. These findings were consistent with those of Shokri et al.^33^. Carbapenem-resistant *A. baumannii* isolates are becoming more common around the world. The majority of these bacteria are highly drug-resistant, including resistance to carbapenems and all other antibiotics, excluding tigecycline and polymyxin^32^. NPs have the potential to be broad-spectrum antibiotics because they can inhibit a wide range of MDR strains that are resistant to most antibiotics^11^. Adeli et al.^34^ discovered that Ag NPs could inhibit pan-drug resistant strains of *Pseudomonas aeruginosa*, *S. aureus*, *E. coli*, and *K. pneumoniae* that were resistant to all antibiotics, including imipenem.

### Patterns of antibiotic resistance and genetic resistance of isolates that produce carbapenemase

Overall, 98% (50/51) of CPGB were resistant to at least three antimicrobial classes and were classified as MDR carriers of resistance genes. According to our findings, *Klebsiella* sp. isolates (6/21) with three resistance genes (10) (*bla*_IMP_, *bla*_OXA_, *bla*_NDM_) (21/50, 42%) had the highest number of XDR (8/21, 38%). PDR was the most common resistance pattern (7/16,43%) among *Pseudomonas* sp. (4/16) isolates showing co-existence of two resistance genes (7) (*bla*_NDM_, *bla*_OXA_) (16/50,32%). In isolates with four resistance genes (8) (*bla*_IMP_, *bla*_OXA_, *bla*_NDM_, *bla*_VIM_) (8/50, 16%), the most common pattern was MDR (4/8,50%) in *A. baumannii* (3/8), isolates with one resistance gene (2) (*bla*_OXA_ and *bla*_NDM_) (5/50, 10%), mostly showed MDR patterns (4/5, 80%) in *Klebsiella* sp., *Pseudomonas* sp*.* and *E. coli* (Table 2).

**Table 2:** Genotypic and phenotypic resistance patterns among CPGB.

Number of carbapenemase genes	Carbapenemase resistance genes	No. of isolates	Resistance profile			Number of common CPGB*	Total no. of isolates (%)
			No. of isolates	%	Type of resistance		
Three genes	*bla*_IMP_*, bla*_OXA-48_*, bla*_NDM_	10	6 8 7	28 38 33	MDR XDR PDR	6 *Klebsiella sp.,* 5 *A. baumannii,* 3 *E. coli,* 2 *P. aeruginosa,2 proteus,* 1 each of, *Serratia,* *Sphingomonas, Pantoea*	21 (42)
	*bla*_VIM_, *bla*_OXA-48_*, bla*_NDM_	9					
	*bla*_IMP_*, bla*_OXA-48,_ *bla*_VIM_	1					
	*bla*_IMP,_ *bla*_KPC_*, bla*_NDM_	1					
Two genes	*bla*_OXA-48,_ *bla*_NDM_	7	3 6 7	18 37 43	MDR XDR PDR	4 *P. aeruginosa,*3 *Klebsiella sp.,* 3 *A. baumannii,* 3 *E. coli,* 1 each of, *Stenotrophomonas, Enterobacter, Morganella*	16 (32)
	*bla*_NDM_, *bla*_VIM_	4					
	*bla*_NDM_*, bla*_KPC_	1					
	*bla*_NDM_*, bla*_IMP_	1					
	*bla*_OXA-48_, *bla*_VIM_	1					
	*bla*_OXA-48_*, bla*_IMP_	1					
	*bla*_IMP_*, bla*_VIM_	1					
Four genes	*bla*_OXA-48_, *bla*_IMP_, *bla*_VIM,_ *bla*_NDM,_	8	4 1 3	50 12 37	MDR XDR PDR	3 *A. baumannii,* 2 *E. coli,* 1 each of, *P. aeruginosa,* *Klebsiella sp., proteus*	8 (16)
One gene	*bla*_OXA-48_	2	4 1 0	80 20 0	MDR XDR PDR	*E. coli* *Klebsiella sp. and P. aeruginosa* *Klebsiella sp.*	5 (10)
	*bla*_NDM_	2					
	*bla*_VIM_	1					
Total no. (%)		50	17	34	MDR		50 (90)
			16	32	XDR		
			17	34	PDR		
*P* value < 0.0001							

CPGB: Carbapenemase-producing Gram-negative bacilli.

Our results are also consistent with the observations of Sadeghi et al.^35^, *K. pneumoniae* strains frequently carry multiple broad-spectrum beta-lactamase enzymes. Single-gene strains have been identified as a major threat to antibacterial chemotherapy^33^. Furthermore, isolates with the greatest number of resistance genes demonstrated greater antibiotic resistance. Isolates carrying three resistance genes, for example, had the highest number of XDR (38%), PDR (33%), and lower MDR (28%) compared to isolates carrying only one resistance gene, which had predominantly MDR (80%) and the lowest XDR (20%), and PDR was not detected. As a result, the development of new antimicrobial agents to combat these resistant isolates is critical. This study found that the isolates were resistant to most antibiotics. There was a significant prevalence of PDR, MDR (34% for both), and XDR (32%) among the 50 MDR isolates carrying multiple carbapenemase resistance genes. Between genetic and antibiotic resistance patterns, a statistically significant association (*P* < 0.0001) was discovered (Table 2).

According to Zendegani and Dolatabadi^32^, carbapenem-resistant *A. baumannii* (100%) carrying resistance genes has a high prevalence of PDR and XDR (*bla*_VEB_, *bla*_PER_, *bla*_OXA51_, *bla*_OXA23_, *bla*_OXA40_, *bla*_IMP_ and *bla*_VIM_). Another study found that of the 91 *P. aeruginosa* isolates tested, 71 (78%) were XDR and 56 (61.5%) were MDR^13^.

Resistance to carbapenems is attributed to the presence of resistance genes, which is consistent with the findings of Algammal et al.^12^ (*bla*_KPC_ and *bla*_NDM_). They discovered that 31.4% of the strains tested were XDR and carried the genes _*bla*OXA_-1, *bla*_CTX_-M, *bla*_TEM_, sul1 and tetA, 22.8% were MDR, and three strains were resistant to carbapenems and shared the genes *bla*_OXA_-1, *bla*_CTX_-M, sul1 and tetA. The high rate of MDR phenotype in this study's CPGBs carrying the carbapenemase gene may be attributed to higher selection pressure due to self-treatment, experimental use, and overuse of third-generation cephalosporins and carbapenems^35^.

Because of their MDR profile, increased incidence, and rapid spread from species to species, and even between different species of gram-negative bacteria via transmissible genetic elements, clinically significant carbapenemase-producing pathogens are considered a triple threat (plasmids, insertion sequences, and transposons). Furthermore, transposable genetic elements contain a large number of antimicrobial resistance genes, which are followed by XDR or MDR traits, which severely limit treatment selection^36^. The ongoing selection of bacteria resistant to various antibiotics has resulted in a renewed emphasis on discovering new, unconventional sources of antibiotics. As a result, the antimicrobial properties of Ag NPs against CPGB were investigated in this study^37^.

### Genotypes of the respective bacterial isolates

**Figure 1:**
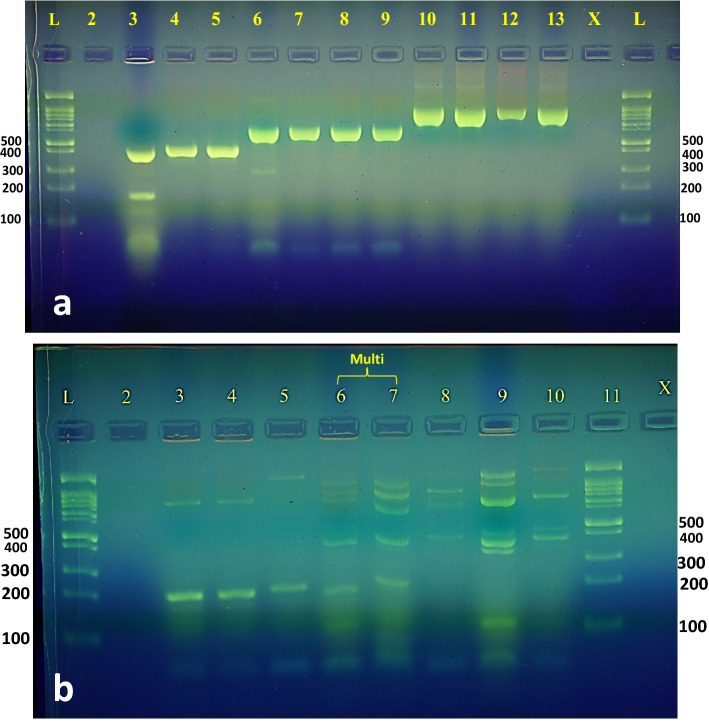
PCR detection of carbapenemase resistance genes KPC, NDM, OXA **(a)** and VIM, IMP **(b)** in CPGB. (**a**) Lanes (3–5), (6–9) and (10–13) imply the OXA-48, NDM, and KPC-positive genes, respectively. (**b**) Lanes (3–5) and (8–10) indicate the IMP and VIM carbapenemase positive genes, respectively, lanes 6 and 7 signify “Multi” coexistence of the VIM and IMP genes. The molecular size of *bla*_NDM_, *bla*_OXA-48_, *bla*_KPC,_ *bla*_VIM_ and *bla*_IMP_ carbapenemase genes tested are 621, 438, and 798, 390 and 232 bp, respectively. Lanes (1,11) in (a) and (1,14) in (b) are 1-kb DNA ladders. Negative control of both figures was in lane 2.

Table 3 shows the results of the PCR analysis of the affected CPGBs. One or more genes were found in 51 of the CPGBs. The most common carbapenemase gene was *bla*_NDM_, which was found in 86% (44/51), followed by *bla*_OXA-48_, which was found in 78% (40/51), *bla*_VIM_, which was found in 50% (26/51), *bla*_IMP_, which was found in 45% (23/51), and *bla*_KPC_, which was found in 7% (4/51) (Fig. 1a and b). The NDM and OXA-48 producers are from 12 different isolates, the IMP and VIM producers are from nine and seven isolates, respectively, and the remaining four KPCs are from three isolates. In general, *Klebsiella* sp. and *A. baumannii* have the greatest number of these genes. Ninety percent (46/51) of the isolates had multiple carbapenemase genes, ranging from two to four. Statistical analysis confirmed that there was a highly significant difference in carbapenemase gene prevalence among CPGB (p < 0.0001) (Table 3).

**Table 3:** Distribution of carbapenemase-encoding genes among CPGB (n = 51).

Bacteria	Number of isolates positive for carbapenemase-encoding genes										Number of isolates positive for multiple genes (2–4)
	IMP		VIM		NDM		OXA-48		KPC		
	No	%	No	%	No	%	No	%	No	%	
*Acinetobacter baumannii*	8	72	5	45	9	81	10	90	2	18	11
*Klebsiella sp.*	1	8	11	91	10	83	8	66	-	-	10
*E. coli*	5	50	4	40	8	80	8	57	1	8	8
*Pseudomonas aeruginosa*	3	37	2	25	7	87	7	78	–	–	7
*Proteus sp.*	2	66	2	66	3	100	3	100	–	–	3
*Enterobacter sp.*	–	–	–	–	1	100	1	100	–	–	1
*Serratia sp.*	1	100	–	–	1	100	1	100	–	–	1
*Stenotrophomonas maltophilia*	1	100	–	–	1	100	1	100	–	–	1
*Sphingomonas sp.*	1	100	–	–	1	100	1	100	–	–	1
*Achromobacter dentrificans*	–	-	1	100	1	100	1	100	1	100	1
*Pantoea agglomerans*	1	100	–	–	1	100	1	100	–	–	1
*Morganella morganii*	–	–	1	100	1	100	1	100	–	–	1
Total no. (%)	23	45	26	50	44	86	40	78	4	7	46 (90%)
*P* value < 0.0001

Similar to previous analyses, *bla*_NDM_ was the most common carbapenemase gene and *bla*_OXA-48_ was the second most common^30,38^. The carbapenemase-producing strain of *K. pneumoniae* was first described in North Carolina in 2001. Although NDM was first identified in India in 2008, it now occurs in a predominant pattern in countless countries around the world^39^.

NDM production in *Enterobacteriaceae*, *Acinetobacter*, and *Pseudomonas aeruginosa* has recently been reported. NDM-producing strains have caused infections resistant to all beta-lactamases, aminoglycosides, nitrofurantoin, quinolones, and sulfonamides. Furthermore, because polygenic NDM codes for chromosomal resistance and plasmid origin, this resistance spreads easily in strains producing NDM^38^. In comparison to previous studies^8,40^, the gene KPC was found in the fewest isolates. Another possibility for the low detection rate of the KPC gene among isolates is that these isolates contain other types of class A carbapenemases (other than KPC)^41^. *Klebsiella* sp. and *A. baumanii* rank first in carbapenemase recovery, which is consistent with data from recent studies^16,29,33^. The production of carbapenemases, such as OXA-48, NDM, and KPC, is well known to be the predominant resistance mechanism among clinical isolates resistant to carbapenem *Enterobacteriaceae* isolates^42^.

*Enterobacteriaceae* that produce carbapenemase and non-fermentative bacilli have long accompanied hospitalised patients. There are numerous reasons for this, such as antibiotic pressure selection or cross-border transport of plasmids containing carbapenemase genes between different *Enterobacteriaceae*^29^. It is important to note that not all carbapenemase-producing isolates are carbapenem-resistant. As a result, carbapenemase production always raises carbapenem MICs but not always sufficiently to be classified as resistant or moderately resistant^42^.

As demonstrated in the current study, a significant proportion of the isolates (46/51) produced more than one carbapenemase gene. These isolates have previously been identified in several studies^29,30,32^. Because of the limited treatment options and the possibility of global spread via horizontal transmission, the coexistence of these carbapenemase genes poses a therapeutic challenge for clinicians. Multiple carbapenemase genes (2–4) have been found in isolates from *A. baumannii* and *Klebsiella* sp. This implies that these microorganisms were able to synthesise a variety of MDR determinants. These strains have previously been described in other publications^13,32,38^.

### Biosynthesis of Ag NPs

**Figure 2:**
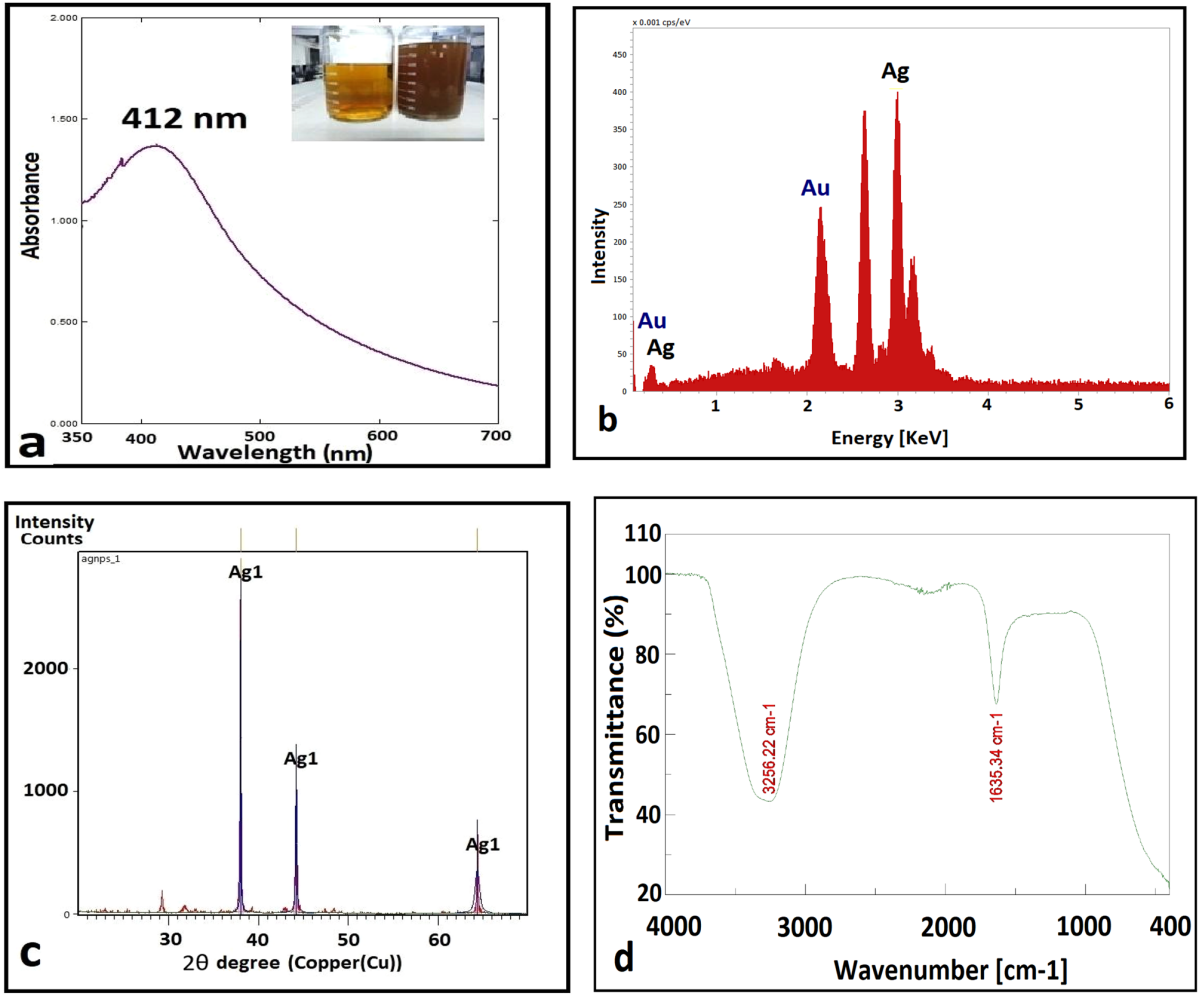
The positive result of color change and UV–visible spectroscopy of biosynthesized Ag NPs **(a),** EDX analysis **(b),** XRD results **(c),** and FT-IR spectra analysis **(d)**.

Within 24 h, the supernatant of *A. baumanii* incubated with AgNO3 solution changed colour from light yellow to dark brown (Fig. 2a), indicating the composition of Ag NPs. When the control mixture (containing no bacteria) was incubated for the same amount of time and under the same conditions, no discoloration was observed.

### Characterization of Ag NPs

One of the most commonly used techniques for NP structural characterisation is UV–visible spectroscopy^10^. The reaction mixture's UV–visible absorption spectra (in the range of 300–800 nm) reveal a strong, single, and intense absorption peak at 412 nm associated with colloidal Ag NPs^21,22^ (Fig. 2a). The colour shift from pale yellow to dark brown was attributed to surface plasmon resonance (SPR)^1,26^. The presence of a single SPR peak indicates that the Ag NPs are spherical in shape^1^. Singh et al.^43^ used *Pseudomonas* sp. for the biosynthesis of Ag NPs, while Pires et al.^5^, Shakir and Shaaban^23^, and Singh et al.^22^, used a similar technique for the biosynthesis of Ag NPs using *A. baumanii,* with absorption spectra reported at 412, 390, and 440 nm, respectively. The precise mechanism underlying the biosynthesis of Ag NPs by bacteria is still unknown. However, it has been reported that metal ions are reduced to form NPs by enzymes such as nitrate reductase, and that these biomolecules are secreted by microbial cells in the matrix, which can act as both reducing and stabilising agents^22^. The absorption band at 400–420 nm is known to be caused by surface plasmon resonance (SPR) in Ag NPs and may be a property of noble metal NPs^43^. In the absorption spectra of spherical metal NPs, a single SPR band would be expected. For Ag NPs, a peak between 410 and 440 nm was observed, as is well known for metallic NPs with sizes ranging from two to 100 nm^22^. In our study, a single SPR peak was observed, indicating that the metal NPs were spherical in shape and parallel to the TEM images. The highest intensity absorption peak of silver at 3 keV was observed in the EDX spectroscopy of Ag NPs (Fig. 2b) confirming the formation of metallic Ag NPs crystalline in nature due to surface plasmon resonance^1,22^. As a result of the Au grid used for EDX analysis, additional Au peaks were discovered. Our XRD results confirm the nanocrystalline structure of biosynthesized Ag NPs. The Ag NPs showed some typical diffraction peaks at 2θ of about 38.037°, 44.205°, and 64.344° (labelled as Ag in Fig. 2c), corresponding to planes 111, 200, and 220, respectively. These diffraction peaks matched the cubically centred faces of the standard silver crystal (JCPDS file no.98–018-0878), as reported in^37,44^. These sharp Bragg peaks could be caused by capping agents that stabilise Ag NP^15^.

**Figure 3:**
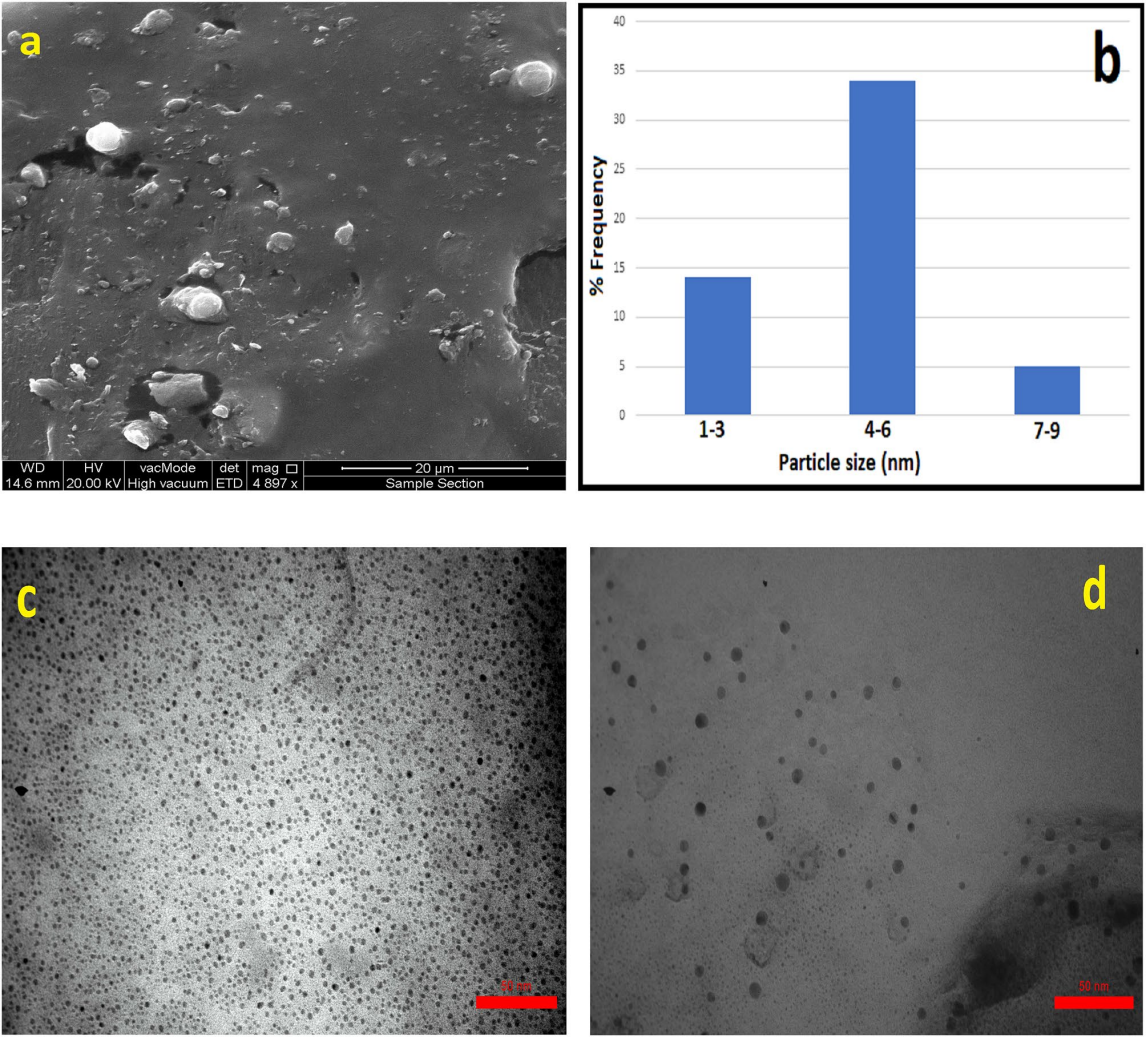
**(a)** FE-SEM analysis**, (c and d)** TEM analysis, and **(b)** Particle size distribution of biosynthesized Ag NPs.

The FT-IR spectroscopy was carried out to confirm potential interactions between silver salts and capping proteins, which could explain the reduction of silver ions and the subsequent stabilisation of Ag NPs (Fig. 2d). Representative spectra of Ag NPs revealed two prominent absorption peaks at 3256.22 and 1635.34 cm^−1^, corresponding to amino group (NH) and carbonyl group (C=O) stretching vibrations, respectively. The presence of amino and carbonyl groups is associated with peptides^1,44^. The FT-IR pattern indicates the presence of capping proteins in the silver solution NP and demonstrates that these proteins are not highly aggregated. The carbonyl groups and free amines of the bacterial proteins may be responsible for the NPs' formation and stability. Protein components in the medium can bind to the NPs via free amino groups or cysteine groups in proteins and the electrostatic attraction of negatively charged carboxylate groups^43^, resulting in the capping of Ag NPs that can help stabilise the NPs in solution and prevent aggregation^14^. The surface morphology of the Ag NPs was studied using FE-SEM. Images from FE-SEM revealed the presence of regularly shaped, spherical Ag NPs (Fig. 3a).

A TEM examination was carried out to better understand the surface morphology and to obtain additional size information. Analysis of TEM images using ImageJ software revealed the presence of several monodisperse, spherical, well-separated, and relatively uniform NPs ranging in size from 1 to 9 nm and with an average size of 4.7 nm (Fig. 3c and d).

The Ag NPs produced by *A. baumannii* appear to be similar to those produced in other studies of *A. baumannii* and *A. calcoaceticus*, where the production of spherical Ag NPs with diameters ranging from 8 to 12 nm has been reported^5,22^. TEM analysis of *Streptomyces xinghaiensis* and *Aspergillus flavus* synthesised Ag NPs revealed the presence of spherical and polydisperse NPs in the 5–20 nm and 5–30 nm size ranges, respectively^10,45^.According to Gurunathan et al.^46^ the size of Ag NP produced by *Allophylus cobbe* leaf extract ranged from two to 10 nm, with an average size of 5 nm.

Fig. 3b depicts a histogram of the percentage age particle size distribution. The particle sizes ranged from 1 to 9 nm, with the following percentage age values: 34% (4 to 6 nm), 14% (1–3 nm), and 5% (7 to 9 nm). Based on the information presented above, it was determined that *A. baumannii* can synthesise small particles (10 nm), indicating a higher antimicrobial potential because the surface area is greater in relation to the volume. Furthermore, there was no evidence of agglomeration in the TEM images. This confirms the stability of the prepared NPs, which was first observed using UV–visible spectroscopy. Small Ag-NPs with uniform distribution have been shown to be effective for a variety of interesting biological activities.

Variations in the shape and size of NPs synthesised by bacterial biosynthetic methods have previously been documented^14,37^. Ag NPs have been developed as promising antimicrobial weapons. Their antimicrobial efficacy is due to their distinct mechanisms of action. Their large surface area, for example, allows for high synergism via polyvalent interactions. Similarly, functional NPs with small molecular ligands exhibit a broad spectrum of antibacterial activity^4^. However, using NP in conjunction with current antibiotics to combat MDR bacterial infections is an alternative strategy^47^.

### Antimicrobial activity of biosynthesized Ag NPs

For severe infections, beta-lactams are frequently the first-line treatment option, with carbapenems serving as a last-resort treatment. As a result, the spread of carbapenem-resistant bacteria is a major public health concern. Infection with these resistant bacteria has a higher mortality rate than infection with carbapenem-susceptible organisms. Efforts have been made to develop new antimicrobial drugs that are resistant to carbapenems^48^.

Ag NPs were tested for antimicrobial activity against the most commonly used XDR, PDR, and MDR strains of *P. aeruginosa*, *E. coli*, *Klebsiella* sp., *A. baumannii*, and *Proteus* sp. antimicrobial susceptibility testing on selected bacteria revealed that these isolates were resistant to ceftriaxone (94%), cefepime (92%), ceftazidime (84%), and imipenem (52%) (Table 1). This study found that Ag NPs had a significant (P < 0.0001) antimicrobial effect on selected CPGB (Table 4).

**Table 4:** Antimicrobial activity of antimicrobials and biosynthesized Ag NPs against CPGB.

Antimicrobial /Ag NPs	MIC (μg/ml)														
	*E. coli*			*Klebsiella sp.*			*Pseudomonas aeruginosa*			*Acinetobacter baumanii*			*Proteus sp.*		
	MIC	MBC	Tolerance ratio	MIC	MBC	Tolerance ratio	MIC	MBC	Tolerance ratio	MIC	MBC	Tolerance ratio	MIC	MBC	Tolerance ratio
Imipenem	8	16	2	16	32	2	8	16	2	8	16	2	16	32	2
Ceftriaxone	≥ 1024	ND		≥ 1024	ND		≥ 1024	ND		≥ 1024	ND		≥ 1024	ND	
Cefepime	≥ 1024	ND		64	128	2	32	64	2	32	64	2	64	128	2
Ceftazidime	≥ 1024	ND		64	128	2	64	128	2	64	128	2	32	64	2
Ag NPs	64	128	2	64	128	2	64	128	2	8	16	2	32	64	2
P-value < 0.0001															

MIC: Minimum inhibitory concentration, MBC: Minimum bactericidal concentration.

The average MIC and MBC of Ag NP were 64, 128 μg/ml against *Klebsiella* sp., *E. coli*, and *P. aeruginosa*, 32, 64 μg/ml against *Proteus* sp., and 8, 16 μg/ml against *A. baumannii,* respectively. In terms of susceptibility, *A. baumannii* was more sensitive to Ag NPs than the other bacteria tested, while *E. coli*, *Klebsiella* sp., and *P. aeruginosa* were less sensitive, which could be explained by their ability to form biofilms^37^.

These findings show that Ag NPs can be used as potential antimicrobial agents and show a broad spectrum of antimicrobial activity. This is an extremely promising result for biosynthesised Ag NPs, which represent a strategy for overcoming bacterial resistance and could be useful in pharmacotherapy.

Ceftriaxone (MIC ≥ 1024 μg/ml) had no effect on growth in any of the isolates tested (Table 4).

The following are the mean MICs of imipenem, ceftazidime, and cefepime, respectively, against the tested species: *E. coli* were 8, 1024, and 64 μg/ml; *Klebsiella* sp., 16, 64, and 64 μg/ml; *P. aeruginosa* and *A. baumanii* 8, 64 and 32 μg/ml; and *Proteus sp*., 16, 32, and 32 μg/ml. All bacteria tested showed significantly higher antibacterial activity than ceftriaxone (1024 μg/ml) and higher bactericidal activity against *E. coli* than ceftazidime and cefepime (1024 μg/ml) (Table 4). The MIC of Ag NPs was significantly lower than that of Singh et al.^22^, who found the MIC of Ag NPs in the range of 150–600 μg/ml against a panel of gram-negative bacteria using *A. calcoaceticus*. Wypij et al.^10^ also reported that Ag NP, which was biosynthesised by *Streptomyces xinghaiensis*, had a high antibacterial activity against *Klebsiella sp.*, *P. aeruginosa* and *E. coli* when compared to commercial antibiotics such as ampicillin, kanamycin, and tetracycline. Ag NPs had MICs of 16, 64, and 256 μg/ml against *P. aeruginosa*, *E. coli* and *Klebsiella sp.*, respectively.

Shaker and Shaban^23^ discovered that *Acinetobacter* Ag NPs had MICs of 3.1, 1.56, and 3.1 mg/ml against MDR *E. coli*, *P. aeruginosa* and *K. pneumoniae*, respectively. In another study, the MIC of Ag NPs was found to be 80 μg/ml for inhibiting carbapenem-resistant and ESBL-producing *P. aeruginosa*^49^. It is well known that the antimicrobial activity of metallic NPs is affected by their shape, size and stability. Smaller NPs have higher microbial toxicity to pathogens because they spread more easily than larger particles^10^. At this point, the Ag NPs are most likely interacting with the cell membrane and can also penetrate the bacteria. The release of Ag + ions is another potential mechanism involved in the antimicrobial activity of Ag NP, which plays a minor but significant role in its bactericidal effect^37^.

### Tolerance determination

Considering the level of tolerance as a measure of the bactericidal potency of an antimicrobial agent (bactericidal agent: MBC/MIC ≤ 2; bacteriostatic agent: MBC/MIC > 2)^26^.

Bactericidal agents kill microorganisms, whereas bacteriostatic agents only inhibit bacterial growth^37^. As the tolerance values were 2, the results in Table 4 suggest that Ag NP has significant bactericidal activity (*P* < 0.0001) against CPGB. Das et al.^26^ and Quinteros et al.^37^ made similar observations. This study demonstrates the utility of biosynthesised Ag NPs as broad-spectrum bactericidal agents against MDR–CPGB. Ag NPs' bacteriostatic and bactericidal activities are due to their ability to degrade the bacterial cell wall, allowing the extrusion of cytoplasmic contents, impeding the respiratory chain, and thus having deleterious effects on DNA^9^.

### Synergistic effect actions of biosynthesized Ag NPs with different antimicrobials

Antibiotic combination therapy is a treatment strategy that is commonly used to treat MDR bacteria. Broad-spectrum beta-lactam antibiotics such as carbapenems and cephalosporins are frequently used in combination with other antibiotics to treat MDR bacteria. We chose these antibiotics to test their potential synergistic effects with Ag NPs. Ag NPs were found to have potent antimicrobial activity against CPGB, with a mean MIC ranging from 64 to 8 μg/ml. All antibacterial combinations with Ag NP demonstrated significant (P < 0.0001) synergistic (FIC ≤ 0.5) and partial synergistic (0.5 < FICI < 1) effects against all bacteria tested. Table 5 shows that the FIC values for the synergy of Ag NP and antimicrobials ranged from 0.13 to 0.5.

**Table 5:** Determination of FIC and combined effect of antibiotics and Ag NPs on strains of CPGB.

Combinations	MIC (μg/ml)																			
	*E. coli*				*Klebsiella* sp.				*Pseudomonas aeruginosa*				*Acinetobacter baumannii*				*Proteus* sp.			
	Ag NPs	Antibiotic	Combined	FIC	Ag NPs	Antibiotic	Combined	FIC	Ag NPs	Antibiotic	Combined	FIC	Ag NPs	Antibiotic	Combined	FIC	Ag NPs	Antibiotic	Combined	FIC
Ag NPs + Imipenem	64	8	4	0.56	64	16	4	0.31	64	8	4	0.56	8	8	2	0.5	32	16	4	0.37
Ag NPs + Ceftriaxone	64	≥ 1024	16	0.26	64	≥ 1024	8	0.13	64	≥ 1024	16	0.26	8	≥ 1024	4	0.5	32	≥ 1024	16	0.51
Ag NPs + Cefepime	64	≥ 1024	16	0.26	64	64	16	0.5	64	32	8	0.37	8	32	2	0.31	32	32	8	0.5
Ag NPs + Ceftazidime	64	≥ 1024	32	0.53	64	64	8	0.25	64	64	16	0.5	8	64	2	0.28	32	32	8	0.5
*P* value < 0.0001																				

FIC: Fractional inhibitory concentration.

Ag NPs show partial synergism FIC = 0.56, 0.53 and 0.51 with imipenem against *E. coli* and *P. aeruginosa*, with ceftazidime and ceftriaxone against *E. coli* and *Proteus sp.*, respectively. No antagonistic activity was observed with any of the combinations. The combination of cefepime plus Ag NPs showed the highest synergistic effect between the combinations compared to other antimicrobials. The CPGBs tested had MICs in the antibiotic resistance range. With the addition of Ag NP, a significant decrease in MIC was observed (*P* < 0.0001) and the bacteria were found to be sensitive to the antibiotics tested (Table 5).

In all isolates tested, the activity of all antimicrobials in combination with Ag NP increased significantly, most notably in the case of *A. baumannii* to ceftriaxone, whose MIC decreased from 1024 to 4 μg/ml. The current study backs up previous findings that Ag NPs are effective bactericidal agents^10,32,46^. These findings are consistent with those of Naqvi et al.^45^, who demonstrated increased efficacy of imipenem, gentamicin, and ciprofloxacin in combination with Ag NP against *P. aeruginosa*, *E. coli* and *K. pneumoniae*, resulting in a 20–35% increase in susceptibility. Antibiotics (tetracycline, ampicillin, and kanamycin) and Ag NP have been shown to have synergistic activity against *P. aeruginosa*, *E. coli* and *K. pneumoniae*^10^.

The MIC of imipenem alone for *A. baumannii* strains ranged from 64 μg/ml to ≥ 256 μg/ml, according to Zendegani and Dolatabadi^32^. In *A. baumannii* strains, Ag NPs conjugated with imipenem increased susceptibility (2 ≤ MIC ≤ 8 μg/ml). It appears to be more effective than NPs and imipenem on their own. Singh et al.^22^ discovered that when Ag NPs were combined with antibiotics, the MICs of *A. baumannii* were significantly reduced.

The combination of antibiotics and Ag NPs may increase binding affinity to their targets, improve penetration through the cell wall barrier, and thus increase the drug's efficacy against bacterial drug resistance. Furthermore, using two antibacterial agents with different bactericidal mechanisms at the same time is important because if bacteria become resistant to one agent, the other antibacterial agent will inhibit the pathogen^32,45^. Because of their small size and large surface area, NPs can carry more drugs, increasing the concentration of antibiotics at the point of contact between the antibiotic and the bacteria, which can further inhibit pathogens. Furthermore, the conjugates contain a lower dose of both antimicrobial agents, which reduces the toxicity to human cells^10,32,45^.

## Conclusions

The appearance of PDR, XDR and MDR in CPGB pathogens constitutes a threat alert indicating the complex therapy produced by these microorganisms. Tigecycline demonstrated promising antibacterial activity against CPGB. Antibiotic resistance was higher in isolates carrying more resistance genes. Biosynthesizing Ag NPs using the *A. baumannii* strain is an eco-friendly, inexpensive, and non-toxic method.

The biosynthesised Ag NPs had a uniform distribution and spherical shape with a diameter of less than 10 nm and demonstrated strong antimicrobial activity. Surprisingly, despite the high rate of antibiotic resistance and the presence of genes encoding carbapenemase resistance, the combination of antibiotics and Ag NPs had a powerful antibacterial effect. The combination therapy can significantly reduce the concentrations of both antibiotics and eliminate the pathogen's antibiotic resistance. These findings imply that biosynthesised Ag NPs can be effective broad-spectrum antibacterial agents, possibly at lower doses than those currently used in clinical trials to treat infections caused by MDR–CPGB isolates.

## Supplementary Information

Supplementary Information.

## Data Availability

The study’s minimal underlying data set has been published in a public data repository on Mendeley and the relevant https://data.mendeley.com/datasets/wm39pzs4wk/1.
