# Supplementary material for: Synergistic antibacterial activity of silver nanoparticles biosynthesized by carbapenem-resistant Gram-negative bacilli
Source: Sci Rep. 2022 Sep 9;12:15254. doi: 10.1038/s41598-022-19698-0 (PMC9463142; doi:10.1038/s41598-022-19698-0)
Supplement: Supplementary file 1 — Supplementary Information. [file 41598_2022_19698_MOESM1_ESM.docx]

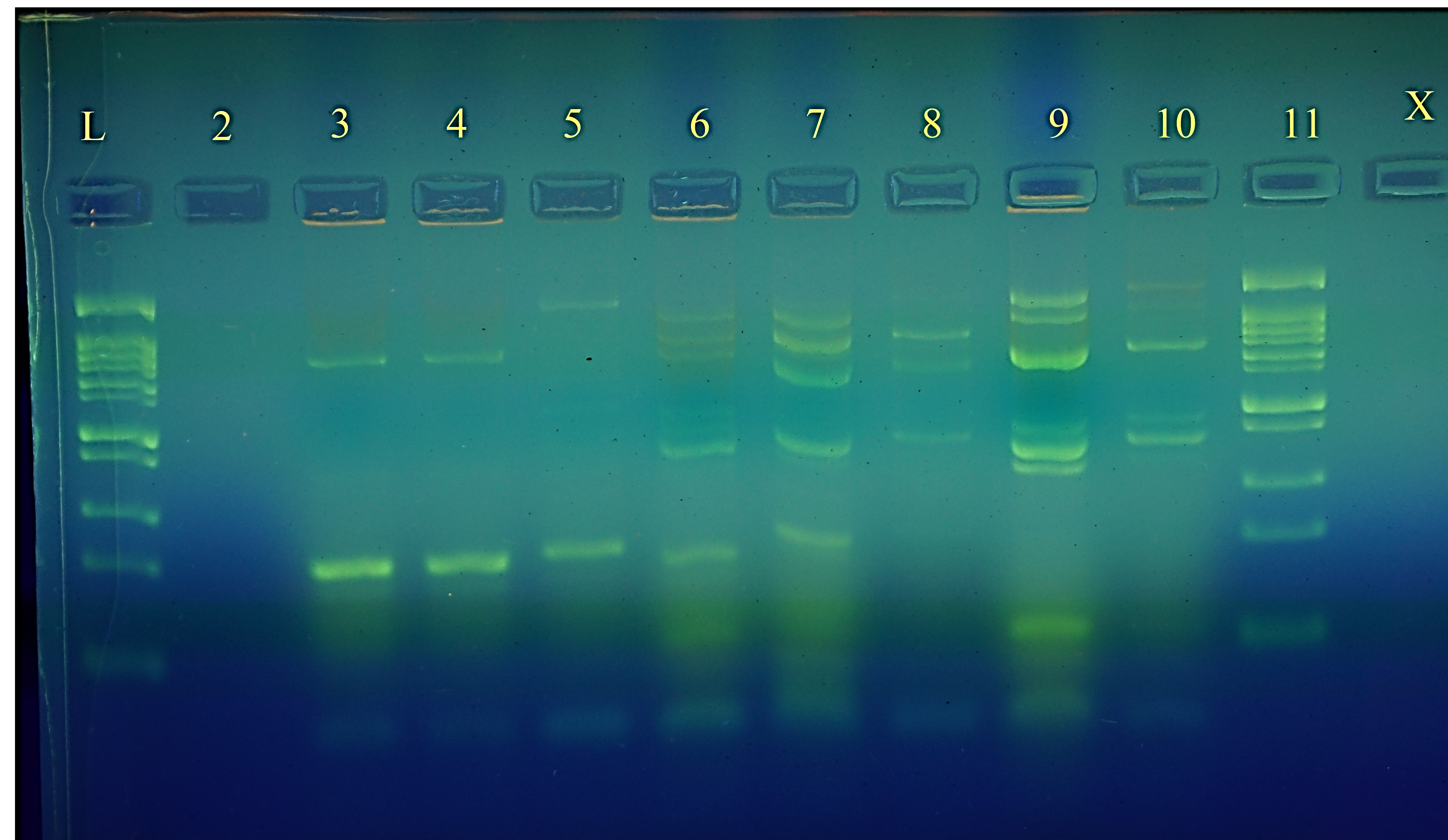


**500**

**200**

**100**

**400**

**IMP (232)**

**100**

**VIM (390)**

**300v**

**Multi**

**Figure 1.b.** Detection of carbapenemase genes IMP, and VIM in carbapenemase-producing Gram-negative bacilli isolates, using multiplex PCR. Lanes 3–5 represent the positive IMP carbapenemase gene, Lanes 8–10 represent the positive VMP carbapenemase gene, Lanes 6 and 7 “multi” represent co-expression of IMP and VIM genes. The molecular size of the IMP and VIM genes are 232 and 390 bp respectively. Lane 1 and 11 are a 1-kb DNA ladder. Lane 2 corresponds to the negative control.
